# Planetary Health: At the Crossroads of CVD Prevention

**DOI:** 10.1007/s11886-025-02272-1

**Published:** 2025-08-14

**Authors:** Tasveer Khawaja, Sanjay Rajagopalan

**Affiliations:** Harrington Heart and Vascular Institute, University Hospitals, Case Western Reserve University, Cleveland, OH USA

**Keywords:** Cardiovascular prevention, Planetary health, Pollution, Climate change

## Abstract

**Purpose of Review:**

To highlight important recent findings demonstrating the interconnectedness of planetary and human health with a focus on cardiovascular disease.

**Recent Findings:**

Data continue to demonstrate a clear interconnectedness between the health of the planet and human health, with cardiovascular disease as an important outcome. The central roles of air pollution, non-optimal temperatures, water/waste management, and food/biodiversity are highlighted. We also highlight the clear opportunity for healthcare organizations to facilitate change that will yield positive environmental and patient outcomes.

**Summary:**

The undeniable interconnectedness of the health of the planet and human health serves as a call to action for physicians, scientists, and policy makers to implement change that will lead to sustainability, a reduction in pollution, and a step into a better future.

## Introduction

Unassailable evidence for the interdependence of human wellbeing and planetary health has necessitated a fundamental shift away from the notion that one’s personal health is distinct from that of the planet. Global surface temperatures were about 1.55 °C above pre-industrial levels (usually defined as the average from 1850–1900) in 2024, with the world experiencing 24 of the hottest years since 2000 [1]. The frequency, intensity, and duration of both unusually high and low temperatures, along with the intensity of natural disasters, including storms, forest fires, and floods, from this time period remains unmatched. From 1998 to 2017, climate-related and geophysical disasters resulted in 1.3 million deaths and affected 4.4 billion people [2]. Given continued global warming and the greater vulnerability of patients with multiple risk factors for CVD, the implementation of change is of paramount importance to curtail these trends [3].

The use of anthropogenic fossil fuel, the large-scale industrialization of food supplies, and land use for human habitation have irrevocably altered the Earth’s ecosystem balances. The Planetary Boundaries framework identifies 9 critical processes essential for maintaining Earth's ecosystem stability and safe human existence [2, 4]. These include climate change, biosphere integrity, land-system change, freshwater use, biogeochemical flows, ocean acidification, atmospheric aerosol loading, stratospheric ozone depletion, and emerging chemical exposures [5]. An analysis in 2023 found that 6 of these 9 boundaries have already been transgressed due to human activities [5]. Violating these boundaries has increased the risk of large-scale abrupt and/or irreversible environmental change, with severe consequences for human health and well-being [5].

Fossil fuels also power 7 key provisioning systems (food, energy, mobility-connectivity, housing, green infrastructure, water, and waste management) [6, 7]. While essential for human living, provisioning systems counterintuitively generate multi-scale risks related to the generation, distribution, and utilization of these inputs in the form of air, water, soil, and chemical pollution, reduced physical activity, lack of green space, and complex geospatial environments that increase stress and reduce social interactions. Further these exposures have been shown to result in the genesis of several risk factors such as hypertension, obesity, type 2 diabetes mellitus (T2D), cancer, dementia, and neuropsychological disorders. Abrupt disruption of provisioning systems, such as during a climate related disaster, may result in food scarcity, power outages, destruction of housing, lack of water, and paralysis of transportation. This educational review will present current evidence of the cardiovascular impact of climate change and fossil fuel pollution with a focus on intersection areas such as food systems, energy, water, waste, and ecosystems/biodiversity. We will discuss the role of health care organizations (HCOs) and sustainability in primary and primordial prevention, and finally conclude with a section on adaptation and mitigation pathways that should be addressed by HCO’s.

## Cardiovascular Health Impact of Climate Change

According to the World Health Organization (WHO), approximately 3.6 billion people live in climate-vulnerable areas. Individuals in climate vulnerable areas may be exposed to a multitude of climate risks, including diarrheal diseases, malaria, dengue, coastal flooding, and temperature extremes [8]. The cardiovascular impact of climate change is hard to precisely estimate, except for factors such as temperature, landscape fires, and other natural disasters, on account of several factors. Firstly, the causal relationship is never direct and frequently involves traditional risk factors such as hypertension, T2D, and obesity. Second, climate change induced alterations in soil nutrition, water quality/pollution, and chemical pollution may have long latency periods that make cause-effect relationships nearly impossible to identify. Thirdly, owing to the multi-system effects of climate change, there are no direct climate specific readouts or biomarkers. Finally, many effects are often embedded in other environmental risk factors, which may further be amplified by social and commercial determinants.

Non-optimal temperatures (NOT), which has strong associations with climate change, presents significant health risks across all periods of suboptimal temperature. According to the Global Burden of Diseases, Injuries, and Risk Factors Study (GBD), 1.17 million (95% CI: 1.07–1.29 million) cardiovascular deaths and 1.81 million (95% CI: 1.65–1.97 million) deaths in 2021 were attributable to NOT [9]. The all-cause disability-adjusted life years (DALYs) due to NOT was 420 per 100,000 (95% CI: 383–461 per 100,000). Excess deaths attributable to elevated temperatures were primarily observed in Europe, while Sub-Saharan Africa had the highest estimates of cold-related excess deaths. Individuals with important risk factors, particularly those suffering from cardiovascular disease, are especially vulnerable, resulting in increased emergency room visits and hospital admissions [10]. Growing and aging populations, urbanization, and evolving socioeconomic development pathways amplify vulnerability to NOT [11]. Air pollution, which originates from the same fossil fuel sources that release greenhouse gases (GHGs), is causally related to cardiovascular disease. Prior estimates have suggested that complete cessation of fossil fuel burning can save 4–5 million lives yearly. Air pollution is also well-known to compound the health impact of high temperatures, leading to more severe outcomes [2, 12]. Increases in landscape fire, including wildfires, have been directly linked to climate change and have been estimated to cause 1.2 million deaths globally.

## Climate Vulnerable Zones and Vulnerability to Health Effects

Climate risks are particularly high for low-income and marginalized populations, especially in countries in the Global South, which have contributed the least to carbon emissions. Over 1.5 billion people in the Global South live in regions where warming exceeds 1.5 °C, especially parts of Africa, South Asia, and Latin America. Countries in sub-Saharan Africa, the Middle East, and South Asia face an eight to tenfold increase in extreme heatwaves compared to the pre-industrial era. Over 30 million climate-related displacements occurred in 2022 alone—90% in the Global South (UNHCR, IDMC data). The Global South’s vulnerability to climate change stems from a mix of geography, poverty, inequity, and power asymmetry [13]. Economically, many of these countries rely on climate-sensitive sectors like agriculture, fishing, and informal labor, with extremely limited social protection or insurance. Infrastructure is typically fragile with many cities lacking basic infrastructure. Access to health care is extremely limited and chronically underfunded, handicapping the ability of many countries to respond to climate disasters. Many countries have no systematic mechanisms for public health surveillance and logistics needed to monitor and respond to climate disasters. Taken together, these factors create extreme vulnerability to the effects of climate change.

## Intersectionality Between Planetary and Cardiovascular Health

**Fig. 1:**
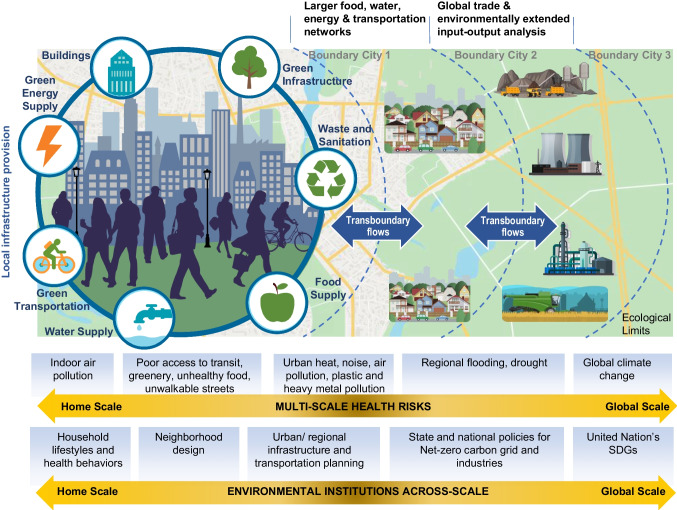
Urban provisioning systems (shown by icons surrounding the City graphic) are decisive for sustainability and health transformation in cities by shaping resource use, impacting exposures and determining health outcomes. (Reprinted with permission from: Rajagopalan S, et al. Circulation. 2024 Apr 9;149(15):e1067-e1089, with permission from the American Heart Association) [17]

The substantial links between cardiovascular and planetary health, including their common drivers, has been discussed previously [14–16]. A Lancet Commission report has recently referred to the shared origins of the epidemics of obesity, malnutrition, and climate change as a syndemic and noted the importance of these to human health and survival [16] A “systems approach” is needed to tackle and understand complex interactions. The interconnectedness of these systems may need to be contextualized and understood before providing solutions. Not doing so may accelerate environmental degradation, perpetuate and worsen inequities, and result in myriad health consequences. Figure 1**,** presents a socio-ecological-infrastructural systems framework, with key provisioning systems of food, energy, water, and waste management [6, 7]. While essential for human living, many provisioning systems are intricately linked and dependent on fossil fuels, generating exposures and risks, such as air pollution. The same provisioning systems are also vulnerable to climate related disasters and may result in acute health risks related to food scarcity, power outages, destruction of housing, lack of water, and paralysis of transportation during a climate-related disaster. By 2050, global demand for food, water, and energy will sharply rise. We will discuss food, water and waste systems that sit at the cross hairs of climate change induced health effects.

### Air Pollution

Climate is a major determinant of air quality and vice versa [18]. The effects of air pollution are attributable to its complex chemistry and size, which are determined by their sources and atmospheric conditions [19–24]. Recent studies have established that particulate matter with a diameter of less than 2.5 µm (PM_2.5_) plus ozone are responsible for 8.4 million excess deaths/year with exposure to PM_2.5_ reducing the global life expectancy by 2.9 years [25, 26]. The ultimate solution to solving air pollution is to shift from fossil fuel to renewable green energy sources. The rate of shift has been substantially slowed with the US cancellation of green energy initiatives, exiting from the Paris agreement, and an explicit reversal of policy decisions to accelerate clean transportation. Although current estimates are unavailable, these measures will undoubtedly have a chilling impact on air pollution levels, which are already increasing owing to the increase in landscape fires in the US and Canada.

Although not without initial cost, the gradual elimination of fossil fuel utilization and reducing the associated anthropogenic air pollution has been shown to have immediate impact on health outcomes [27]. It has been shown that much of the economic benefit that comes from decarbonization is driven by reductions in air pollution related health costs [28]. More than half of the immediate health impact of air pollution reduction may relate to reduction in cardiovascular mortality and reduced hospitalizations [12]. The impact on susceptible populations, particularly racial minorities and the socially disadvantaged that contribute disproportionately to cost of care, may indeed be quite substantive. Furthermore, there are several potential synergies that may result from decarbonization [29]. These include: (1) Adoption of public or active forms of travel together with electrification of transportation, yielding reduced noise pollution and increased physical activity; (2) decarbonization in the agricultural sector will lead to reductions in cardiovascular disease due to increased intake of locally sourced and sustainable plant-based ingredients; (3) the prevalence of important risk factors, such as hypertension, obesity, and T2D, will decrease due to reductions in chemical exposures due to elimination of petroleum-based products.

### Landscape Fires (LFS) and Dust storms

Landscape fires include wildfire disasters, prescribed burning, agricultural burning, and deforestation fires [30]. All can have substantial impacts on human health and well-being through exposure to heat, emissions, and altered ecosystem functioning (e.g., biodiversity, water quality, etc.). The global burden attributable to landscape fire smoke between 2000–19, has been estimated at 1.53 million all-cause deaths per year, including 0.45 million cardiovascular deaths and 0.22 million respiratory deaths [31]. Over 90% of all attributable deaths were in low-income and middle-income countries. Climate change has increased the frequency of dust storms, with wildfires and heat waves often occurring together. Several reviews have covered the expanding number of studies and meta-analysis that have linked LFS with acute myocardial infarction, stroke, and hospitalizations [32–34]. Reducing the short- and longer-term health harms associated with LFS requires reducing the genesis of these episodes. The use of prescribed burning involves complex trade-offs between the risks and benefits of smoke exposure, which will vary according to topography, fire history, vegetation, along with population distribution and vulnerability [30]. The greatest opportunity for reducing the large global burden of disease of LFS lies with changing the management of agricultural burning and tropical deforestation fires. For example, nonburning alternatives to agricultural waste management have the potential to substantially reduce pollution, store carbon, and generate income [35].

### Temperature

Both low and high temperatures contribute to cardiovascular morbidity and mortality [36, 37]. Globally, ~ 5 million deaths were attributable to NOT per year, accounting for 9.43% of all deaths; 8.52% were cold-related and 0.91% were heat-related [3]. A substantial proportion of this is related to cardiovascular events. The mechanisms related to heat induced CVD relate to increased hemodynamic demand, dehydration, inflammation, heightened thrombosis, and increased sympathetic tone. Meta-analyses also confirm that atherosclerotic cardiovascular disease is the leading cause of death with NOT [3, 14]. In the large multi-city collaborative (MCC) network involving 567 cities from 27 countries, the relationship between temperatures and ischemic heart disease events was non-linear [38]. The pooled RRs of death associated with extreme heat (99th percentile vs MMT) in this study from ischemic heart disease, stroke, and heart failure were 1.07 (95% CI, 1.04–1.10), 1.10 (95% CI, 1.06–1.15), and 1.12 (95% CI, 1.05–1.19), respectively. The concept of “climate penalty” refers to the enhanced formation of secondary pollutants, such as ground-level ozone, with rising temperatures and altered meteorologic conditions caused by climate change. The link between cardiovascular mortality and ozone levels has been clearly established [39–41]. A time-series study in 2017 examined the 2-way effect modifications of higher air temperature and pollution in 8 European cities. On days with higher air temperature, air pollution had a stronger association on total and cardiovascular disease related mortality [42]. Similarly, a greater risk of heat-related CVD mortality is observed in low-middle-income countries (LMICs) compared to upper-middle-income countries and high-income-countries (HICs). These countries often experience high summer temperatures and intense heatwaves, exacerbating vulnerability due to inadequate infrastructure, such as a lack of electricity, air conditioners, and efficient healthcare services during heatwaves [43]. Many of the most frequently prescribed medications, such as anticholinergics, antihypertensives, antiarrhythmics, antianginals, diuretics, antidepressants, insulin, and certain analgesics, have heat-related risks, including reduced effectiveness and adverse side effects, such as decreased sweating [37]. To reduce urban heat island effects, cities can adopt a range of nature-based, architectural, and policy-driven interventions. These include expanding tree cover, installing green roofs, using reflective building materials, promoting permeable and cool pavements, and incorporating water features [44]. Smart urban planning—like designing shaded streets, improving ventilation corridors, and enhancing energy efficiency—also plays a vital role. These strategies not only lower ambient temperatures but also improve air quality, reduce noise, and promote physical activity by creating more walkable environments. The cardiometabolic benefits are significant: lower heat exposure reduces cardiovascular strain and dehydration risk; better air quality reduces inflammation and vascular injury; and greener, cooler urban spaces encourage physical activity and social cohesion, all of which support healthier blood pressure, glucose regulation, and mental well-being.

### Food Systems and Biodiversity Loss

About 11 million premature deaths, mostly due to ASCVD, are annually linked to unhealthy diets [45]. In 2018, a total of 14.1 million (95% UI: 13.8, 14.4 million) estimated new T2D cases, or 70.3% (95% UI: 68.8–71.8%) of the total, were estimated to be due to suboptimal intake of the 11 dietary factors [46]. Excess intake of six harmful dietary factors jointly (refined rice and wheat, processed meats, unprocessed red meat, sugar sweetened beverages, potatoes, fruit juice) contributed a larger proportion of the total global diet-attributable burden (60.8%) than insufficient intake of five protective dietary factors (whole grains, yogurt, fruits, non-starchy vegetables, nuts and seeds; 39.2%) [47]. Factors that ultimately lead to increased consumption of harmful foods are complex and encompass not only availability and quality of food, but also the policies, economic forces, and environmental factors that influence food production, distribution, and access. A 2019 Lancet commission report emphasized the urgent need to transform our global food system to achieve sustainable development and climate goals and advocated for a shift towards healthy, plant-based diets, and sustainability [48].

Policy changes and incentives can reverse the impact on current food practices. Federal policies that incentivize the food supply chain to increase supply and decrease cost of nutritious foods, taxing sugar sweetened beverages, innovative food system policies directed at private retailers, and discourage unhealthy foods with default healthy choices (e.g., no SSBs in children’s meals, smaller portion sizes, whole grain rather than refined grains in breads/rolls) would be effective [49].

### Water and Waste Systems

The water supplies of many urban areas of the United States and across the globe face incursion from ubiquitous chemical and trace metal exposures. The latter includes lead, nickel, cadmium and arsenic, which are all associated with cardiometabolic disease [12, 50]. The recent explosion of metals such as cadmium and nickel in batteries, plastics and plastic-related chemicals such as phthalates and legacy chemicals, including bisphenol A (BPA), bisphenol S, per- and polyfluoroalkyl substance (PFAS) have already penetrated water systems [51, 52]. Most urban water management systems in the United States do not purify chemicals and metals and thus, these concerns demand the transformation of urban water systems from source to use [44]. Data from the National Health and Nutrition Examination Survey demonstrate the ubiquity of PFAS exposure [53]. Recent evidence suggests strong evidence linking a variety of PFAS with cardiovascular outcomes [53].

The role of solid waste management in achieving sustainable development is emphasized in several international development agendas [54]. Uncollected and untreated waste has environmental impact, including methane emissions, air pollution, and land/water contamination with chemicals that potentiate CVD [12]. The decomposition of biodegradable waste under anaerobic conditions contributes to about 18% of global methane emissions [55]. Open waste burning and dumping is common in many urban cities of the Global South [56]. These practices almost always affect marginalized social groups near the disposal sites. An integrated approach to waste and water that emphasizes improving all stages, including procurement, treatment, transfer/sorting, and disposal must be grounded on sustainability [57].

## Health Care Organization (HCO) Role in Climate Change

Healthcare systems are significant contributors to environmental pollution and GHGs [12, 58, 59]. The mortality rate from US healthcare pollution is estimated to be comparable to that of errors during delivery of healthcare [60, 61]. In an analysis for 189 countries, 4.4% of GHGs, 2.8% of PM_2.5_, 3.4% of NO_x_, and 3.6% of SO_2_ was attributable to health care activities [58]. There is substantial variation by country with China, US, and India generating the highest total PM_2.5_ footprints [62]. Given the direct impact of anthropogenic emissions on health, and the moral high ground of HCOs, they could lead the way in emissions control.

## Reducing the Environmental and Carbon Footprint of Cardiac ICU and Health Systems: A Pivot to Sustainability in Healthcare

Focusing on carbon emissions alone, without appropriate incentives for behavior change, is unlikely to accelerate the transition to net zero emissions [63]. There continues to be a lack of understanding of the links between carbon emissions and environmental health and appreciation of the concept that what is good for the environment and planet is also good for health [64]. The adoption of sustainable practices encompasses three key drivers which also serve as impact areas: economic, environmental, and social practices. Placing the economic argument at the forefront of sustainability in health care can provide the appropriate incentive to influence the other two pillars. Countries worldwide are experiencing shortfalls in funding for healthcare, that may not allow prioritization of resources for climate-change efforts unless an economic argument can also be made [65].

### Cardiovascular Prevention as a Core Precept for Sustainability

Cardiovascular prevention can be recast to not only improve cardiovascular outcomes, but as a means of = optimizing both human and environmental health through sustainable practices. Primordial and primary prevention decreases procedural interventions, hospital admissions, clinic visits, and medical devices and collectively lower emissions and resource consumption. Upholding other important behaviors, such as harnessing plant-based planetary diets, physical activity, smoking cessation, and self-care, motivates patients to take ownership of their heart health, while emphasizing sustainability.

### Reducing the Carbon and Environmental Footprint of ICUs

The carbon footprint of the ICU in the modern era is quite considerable. This is primarily driven by energy harnessed for several essential tasks, including heating, ventilation, and powering essential electrical equipment. In contrast, plastic use, nutrition, laundering, ventilators, and other ICU processes and machines contribute to comparatively less emissions [66]. Identification of areas for resource optimization in the operating room and ICU based on local practices and efficient practices is an important strategy to reduce waste/decease cost [67]. Procedures come with considerable cost in the form of significant material consumption, which is associated with a carbon and environmental cost [68]. Customizing procedure kits to reduce waste and redundancy, maintain only essential device inventory, and allowing for equipment use beyond expiration dates when appropriate are all key. Ensuring renewable power is used for both the lab and the washing and sterilizing of equipment, minimizing single-use items, implementing recycling programs, installation of energy-efficient equipment and lighting, reducing power utilization during downtimes, and eliminating paper usage will yield substantial reductions in carbon footprints [68].

## Hospital Food and Cafeteria Practices

Reducing food waste in hospitals cafeterias and fossil fuel use for transportation are likely to be effective, but currently suffer from appropriate incentive models [69, 70]. It has been demonstrated that when = hospital staff make healthy life choices (exercising regularly, eating healthy, etc.), patients are more likely to incorporate guidance regarding these lifestyle choice. Training health care personnel to not only demonstrate competencies in nutrition, but an understanding of sustainable foods and the health and carbon impact of dietary decisions may help create a transformative and conscious workforce. Healthcare workers who are often exposed to less-than-optimal work environments, will also benefit from the healthy impact of such choices [71].

## Health Care Organization Role in Climate Resilience Adaptation

The health system is crucial in addressing climate impacts due to its role in managing public health risks, disease prevention, emergency response, and care for vulnerable populations [29, 72]. The WHO Health System Resilience and Operational Framework for Climate Resilient and Low Carbon Health Systems highlight key areas for [73, 74] sustainable financing, governance and leadership, health workforce and service delivery, health information systems, medical products and technologies, community engagement, and reduction in carbon emissions. Resilient health systems can help absorb and adapt to the challenges posed by climate change, while ensuring essential health services to high-risk or vulnerable populations and promoting sustainable well-being. Yet, few countries have begun implementing such resilience and adaptation plans. Below are some of the measures to improve health system resilience and minimize climate-related health hazards.

### Health Policy Measures, Research, and Training

Policies for heat risk adaptation, coastal protection, and creating national databases for climate actions, all aimed at ensuring universal healthcare access are needed. Unfortunately, most countries, especially in the developing world, at risk for climate-related health impacts lack detailed policy measures. Effective climate change mitigation and adaptation depends on long-term planning, collaboration with non-health sectors, sufficient funding, regular policy evaluation, and enforcement of regulations.

### Infrastructure and Supply Chain Management

Responses to climate change also include upgrading health infrastructure, ensuring medical equipment resilience, and establishing backup power sources for uninterrupted healthcare during climate events. Improving ventilation for thermal comfort and enhancing health supply chains by stockpiling essentials are also key strategies. The implementation of early warning systems designed to alert health systems of impending climate-sensitive diseases is key.

### Community Engagement, Equity, and Integrating Primary Care

Community engagement allows health systems to build their social capital and resilience at the individual and community levels to reduce climate-related health costs. Strengthening primary healthcare, improving, governance, fostering multisectoral coordination, and tailoring strategies to local contexts enhances adaptability to address emerging challenges.

### Health Promotion and Communication of Climate Risks

Educating both communities and healthcare professionals about the health impacts of climate change, environmental pollution, and the co-benefits of mitigation is essential. To implement this, climate-informed health education programs may serve as a key resource. Effective adaptation also involves emergency risk communication, particularly for high-risk populations, health messaging during extreme events, and rapid, disease-specific emergency responses.

### Occupational Health and Safety and One Health

Integrating climate change effects into Occupational Health and Safety (OHS) assessments and increasing research on these implications are crucial for adapting health systems to climate change. Protecting healthcare workers involves mitigating heat stress, improving ergonomics, enhancing staffing and insurance, and refining work processes. Beyond the WHO framework, which focuses on governance and service delivery, additional strategies, including social support systems, One Health approaches, which emphasize the interconnectedness of human health, animal health, and the environment, are much needed.

## Conclusions

NOT and climate hazards disproportionately affect those with cardiovascular, respiratory, and kidney diseases. Vulnerable groups—especially individuals with pre-existing CVD, face heightened risks of acute cardiovascular events during heatwaves. These risks are further exacerbated by age, socioeconomic status, and environmental conditions, like air pollution. As global temperatures climb, the frequency and intensity of heatwaves are expected to increase, which will pressure the body's thermoregulatory responses to heat stress and strain the cardiovascular system, especially in individuals with existing health issues. Heatwaves are linked to increased incidences of ischemic heart disease, strokes, heart failure, and arrhythmias. Effective public health strategies, such as heat-health action plans recommended by WHO, are crucial to reduce these risks. These plans should include early warning systems, education on recognizing heat-related symptoms, adjusting medications during heatwaves, and preparing ICUs for these specific challenges. Climate adaptation and mitigation plans are going to be critical in reducing morbidity and mortality in response to non-optimal temperature and extreme events.

## Key References

- Rajagopalan S, Landrigan PJ. Pollution and the Heart. N Engl J Med 2021;385(20):1881–1892. (In eng). 10.1056/NEJMra2030281.
  - This reivew article highlights the important links between cardiovascular disease and pollution. This includes different forms of pollutants and mechanisms.
- Swinburn BA, Kraak VI, Allender S, et al. The Global Syndemic of Obesity, Undernutrition, and Climate Change: The Lancet Commission report. Lancet 2019;393(10,173):791–846. 10.1016/S0140-6736(18)32822-8.
  - This report highlights the shared origins of malnutrition, including obesity and undernutrition, and climate change under the framework of a syndemic.
- WHO. Health system resilience framework. 2015. Available at: https://www.who.int/publications/i/item/9789241565073. Accessed on August 14, 2024.
  - This document highlights the WHO’s recommendations for building a healthcare system that is resilient in the face of climate change.

## Data Availability

No datasets were generated or analysed during the current study.
